# New Antimicrobial Gels Based on Clove Essential Oil–Cyclodextrin Complex and Plant Extracts for Topical Use

**DOI:** 10.3390/gels11080653

**Published:** 2025-08-18

**Authors:** Alina Ionela Stancu, Lia Mara Dițu, Eliza Oprea, Anton Ficai, Irinel Adriana Badea, Mihaela Buleandră, Oana Brîncoveanu, Anca Gabriela Mirea, Sorina Nicoleta Voicu, Adina Magdalena Musuc, Ludmila Aricov, Daniela Cristina Culita, Magdalena Mititelu

**Affiliations:** 1 Department Science and Engineering of Oxide Materials and Nanomaterials, Faculty of Chemical Engineering and Biotechnology, National University of Science and Technology Politehnica Bucharest, 1–7 Polizu Street, 011061 Bucharest, Romania; alina.stancu1995@gmail.com; 2 Department of Botany and Microbiology, Faculty of Biology, University of Bucharest, Portocalilor 1–3, 060101 Bucharest, Romania; lia-mara.ditu@bio.unibuc.ro; 3 Academy of Romanian Scientists, Ilfov Street 1–3, 050045 Bucharest, Romania; 4 Department of Analytical Chemistry and Physical Chemistry, Faculty of Chemistry, University of Bucharest, 90–92 Panduri Street, 050663 Bucharest, Romania; irinel.badea@chimie.unibuc.ro (I.A.B.); mihaela.buleandra@g.unibuc.ro (M.B.); 5 National Institute for Research and Development in Microtechnologies, 126A Erou Iancu Nicolae Street, Voluntari City, Ilfov County, 077190 Bucharest, Romania; oana.brincoveanu@imt.ro; 6 National Institute of Materials Physics, 405A Atomistilor Street, 077125 Magurele, Romania; anca.coman@infim.ro; 7 Department of Biochemistry and Molecular Biology, Faculty of Biology, University of Bucharest, 030018 Bucharest, Romania; sorina.voicu@bio.unibuc.ro; 8 ”Ilie Murgulescu” Institute of Physical Chemistry, 202 Spl. Independentei, 060021 Bucharest, Romania; amusuc@icf.ro (A.M.M.); laricov@icf.ro (L.A.); dculita@icf.ro (D.C.C.); 9 Department of Clinical Laboratory and Food Safety, Faculty of Pharmacy, University of Medicine and Pharmacy Carol Davila, 6 Traian Vuia Street, 020956 Bucharest, Romania; magdalena.mititelu@umfcd.ro

**Keywords:** antimicrobial agents, *β*-cyclodextrin complex, *Eugenya caryophyllata*, *Verbena officinalis*, *Aloysia triphylla*, *Laurus nobilis*, alginate, chitosan, Carbopol 940

## Abstract

This study aimed to develop and characterise novel hydrogels based on natural bioactive compounds for topical antimicrobial applications. Four gel systems were formulated using different polymers, namely polyacrylic acid (Carbopol 940, CBP-G), chitosan with high and medium molecular weights (CTH-G and CTM-G), and sodium alginate (ALG-G), incorporating tinctures of *Verbena officinalis* and *Aloysia triphylla*, *Laurus nobilis* essential oil, and a *β*-cyclodextrin–clove essential oil complex. All gels displayed a homogeneous macroscopic appearance and maintained stability for over 90 days. Rheological studies demonstrated gel-like behaviour for CBP-G and ALG-G, with well-defined linear viscoelastic regions and distinct yield points, while CTM-G exhibited viscoelastic liquid-like properties. SEM imaging confirmed uniform and continuous matrices, supporting controlled active compound distribution. Thermogravimetric analysis (TG-DTA) revealed a two-step degradation profile for all gels, characterised by high thermal stability up to 230 °C and near-total decomposition by 500 °C. FTIR spectra confirmed the incorporation of bioactive compounds and products and highlighted varying interaction strengths with polymer matrices, which were stronger in CBP-G and CTH-G. Antimicrobial evaluation demonstrated that chitosan-based gels exhibited the most potent inhibitory and antibiofilm effects (MIC = 2.34 mg/mL) and a cytocompatibility assessment on HaCaT keratinocytes showed enhanced cell viability for chitosan gels and dose-dependent cytotoxicity for alginate formulations at high concentrations. Overall, chitosan-based gels displayed the most favourable combination of stability, antimicrobial activity, and biocompatibility, suggesting their potential for topical pharmaceutical use.

## 1. Introduction

The skin is the body’s primary protection against environmental threats such as chemicals, heat, and pathogens. When this barrier is compromised, as frequently occurs in cases of burns or skin infections, there is a significant increase in the risk of both local and systemic infections, which delays healing. Burn injuries, particularly those of the second and third degrees, have been shown to cause tissue damage and weaken the skin’s defences [1,2,3]. This creates an ideal medium for the growth of opportunistic pathogens (such as *Acinetobacter baumannii*, *Pseudomonas aeruginosa*, and *Staphylococcus aureus*), sometimes multidrug-resistant strains, and it could result in delayed epithelialisation, persistent inflammation, or sepsis. Similarly, cutaneous infections are prevalent, encompassing both minor ailments such as impetigo and folliculitis, and more severe conditions, including cellulitis and infected ulcers. Consequently, developing broad-spectrum antimicrobial topical formulations is crucial because they offer the advantage of delivering therapeutic agents directly to the action site, minimising systemic side effects while fostering a moist and protective wound environment. Semisolid formulations, often polymer-based, are ideal for this purpose due to their biocompatibility and flexible properties (e.g., pH, viscosity).

Hydrogels, which are characterised by their high water content and skin compatibility, are experiencing a surge in utilisation for the treatment of burns and infected skin lesions. The incorporation of antimicrobial agents such as metal nanoparticles, essential oils, antibiotics, and plant compounds is facilitated by their adaptable structure. Recent advancements in hydrogel technology have focused on the use of polymer carriers (e.g., cyclodextrins, biodegradable polymers) to improve the stability, bioavailability, and controlled release of these active ingredients. This research aims to enhance therapeutic efficacy while minimising the risk of resistance and cytotoxicity. These polymers vary in their molecular weight and crosslinking degree depending on their specific type, and are well suited for applications on the skin or mucosal membranes [1,2,3,4].

However, the porous nature of hydrogels could lead to rapid diffusion and premature release of small-molecule active pharmaceutical ingredients if they are not properly integrated. The rheological properties (viscosity and consistency) of hydrogels could also be affected by the administration route, environment, and physicochemical properties of incorporated compounds, potentially compromising the structural integrity of the polymer matrix [5,6,7,8,9,10,11,12].

In the realm of biomedical applications, sodium alginate and chitosan, being naturally derived polysaccharides, have garnered considerable attention due to their advantageous properties. Sodium alginate, a natural polymer extracted from brown seaweed, has emerged as a material of choice due to its biocompatibility, non-toxicity, and mild gelation properties. The formation of stable, porous hydrogels is achieved through ionic crosslinking with divalent cations, such as Ca^2+^, resulting in optimal controlled release of bioactive compounds. In topical formulations, alginate’s high absorbency, capacity to sustain a moist wound environment, and haemostatic properties render it suitable for use in burn dressings and infected wound care. Moreover, the encapsulation of antimicrobial agents within the polymeric network has been demonstrated to enhance their stability and topical action [13,14].

In addition, chitosan, a cationic polysaccharide derived from chitin, is recognised for its biocompatibility, biodegradability, and inherent antimicrobial properties. The mucoadhesive nature of the substance, combined with its ability to form hydrogels under mild conditions, makes it suitable for topical applications where prolonged retention is essential. The incorporation of chitosan hydrogels into antimicrobial formulations has been demonstrated to enhance the efficacy of such treatments by interacting with microbial membranes. Furthermore, its potential for combination with other antimicrobial agents, its ability to form a film on the skin, and its capacity to modulate inflammation have been proven, all of which support its use in treating skin infections and promoting tissue regeneration [15,16].

Another biopolymer with interesting properties is polyacrylic acid (Carbopol). It is derived from acrylic acid and forms transparent, stable hydrogels with adjustable rheological properties through neutralisation with alkaline agents such as sodium hydroxide or triethanolamine. Due to its excellent skin tolerance and ease of formulation, Carbopol is widely used in topical semisolid formulations, particularly for drug delivery systems that require precise control over viscosity and release kinetics. Though lacking intrinsic antimicrobial activity, Carbopol hydrogels serve as ideal matrices for incorporating active agents, enabling sustained release and improved local bioavailability [17,18].

Furthermore, a promising approach for enhancing gel textures and rheological properties is the use of cyclodextrins in hydrogel formulation due to their ability to form inclusion complexes with bioactive compounds, including essential oils. Thus, complexation offers these volatile molecules protection against environmental degradation, evaporation, and adverse conditions such as heat and light. Consequently, cyclodextrin-based delivery systems have gained significant attention as efficient carriers for bioactive agents [19,20].

Many medicinal plants have a chemical composition that makes them attractive for their therapeutic properties and potential use in drug development. Their diverse array of bioactive compounds, such as alkaloids, flavonoids, terpenes, tannins, and essential oils, often exhibits a wide range of pharmacological activities. Thus, phytochemical analyses of *Verbena officinalis* and *Aloysia citrodora* (*Verbenaceae* family), known for their medicinal properties, reveal various bioactive compounds, particularly abundant flavonoids and terpenoids [21,22]. Their high content of phenolic compounds contributes to the reported antimicrobial effects of their alcoholic extracts, exhibiting growth-inhibitory activity against pathogens such as *Staphylococcus aureus*, *Salmonella* spp., *Escherichia coli*, *Pseudomonas aeruginosa*, *Bacillus subtilis*, and *Candida* spp. [23,24,25,26].

Another species traditionally used in herbal medicine is the Mediterranean shrub, *Laurus nobilis* L. (bay leaf). Recent studies highlighted the significant antimicrobial properties of *L. nobilis*, primarily attributed to its essential oil and phenolic compounds. The essential oil of *L. nobilis*, rich in 1,8-cineole, demonstrated antimicrobial activity against various bacteria (e.g., *Staphylococcus aureus*, *Escherichia coli)* and fungi, also inhibiting biofilm formation, indicating its broad-spectrum antimicrobial potential [27,28,29,30].

Based on our previous findings regarding the chemical composition and antimicrobial properties of *Eugenia caryophyllata* essential oil and its inclusion complex with *β*-cyclodextrin [31], as well as similar results obtained for tinctures from two plants in the *Verbenaceae* family [32], the present study aims to formulate and characterise new topical gel systems formulated using different polymers, incorporating tinctures of *Verbena officinalis* and *Aloysia triphylla*, *Laurus nobilis* essential oil, and a *β*-cyclodextrin–clove oil complex. These new hydrogels were characterised using various methods to gain a better understanding of their structural, thermal, and flow-related properties, and their antimicrobial activity was tested against different bacterial strains. Additionally, this study aimed to enhance the stability and bioactivity of the mentioned natural extracts, to which free volatile oil of *Laurus nobilis* was added, thereby developing novel topical formulations with improved therapeutic efficacy.

## 2. Results and Discussion

### 2.1. Chemical Characterisation of the Plant-Derived Ingredients

#### 2.1.1. The Inclusion Complex’s Chemical Composition

The *β*-cyclodextrin–*Eugenia caryophyllata* (clove) volatile oil complex was a compact white powder with a pronounced clove-like aroma. The effectiveness of *β*-cyclodextrin in capturing volatile components was demonstrated by its high encapsulation efficiency (83–35%), which means approximately 170 mg of volatile oil per gram of the complex. The most abundant constituents (established by GC-MS analysis) were eugenol (90.67%), followed by eugenyl acetate (4.77%) and (E)-*β*-caryophyllene (3.98%), among other compounds, as detailed in an analysis and comprehensive characterisation already published recently [31].

#### 2.1.2. Tincture Chemical Characterisation

An Ultra-High-Performance Liquid Chromatography with Diode Array Detection and Mass Spectrometry (UHPLC-DAD/MS) analysis previously published (which included a complete chemical characterisation of these extracts) indicates that *Verbena officinalis* (VOT) and *Aloysia triphylla* tinctures (ATT) contain an extensive range of phenolic compounds, including phenolic acids, flavonoids, and flavonoid-based heterosides (e.g., naringin, rutin, and hesperidin), as well as trace amounts of resveratrol. While p-coumaric acid was the most abundant compound in ATT, followed by ferulic acid, 4-hydroxybenzoic acid, and caffeic acid, in VAT, apigenin was the main compound, followed by syringic acid, 4-hydroxybenzoic acid, ferulic acid, caffeic acid, and cinnamic acid [32].

#### 2.1.3. Gas Chromatography–Mass Spectrometry (GC-MS) Analysis Results of *Laurus nobilis* Essential Oil

The GC-MS chemical composition of *Laurus nobilis* (bay) essential oil, as shown in Table S1, includes a high eucalyptol content, which agrees with the approximate 70% reported by Basak et al. (2013) [33].

### 2.2. Physical Characterisation of the New Gels

#### 2.2.1. Macroscopic Characteristics

The characteristics of the gel samples, as presented in Tables S2 and S3, indicate that both the newly formulated gels and their bases are homogeneous. All gels exhibited good stability, maintaining a uniform appearance at 2–8 °C, as well as at room temperature, after 90 days.

#### 2.2.2. Rheological Data

**Figure 1:**
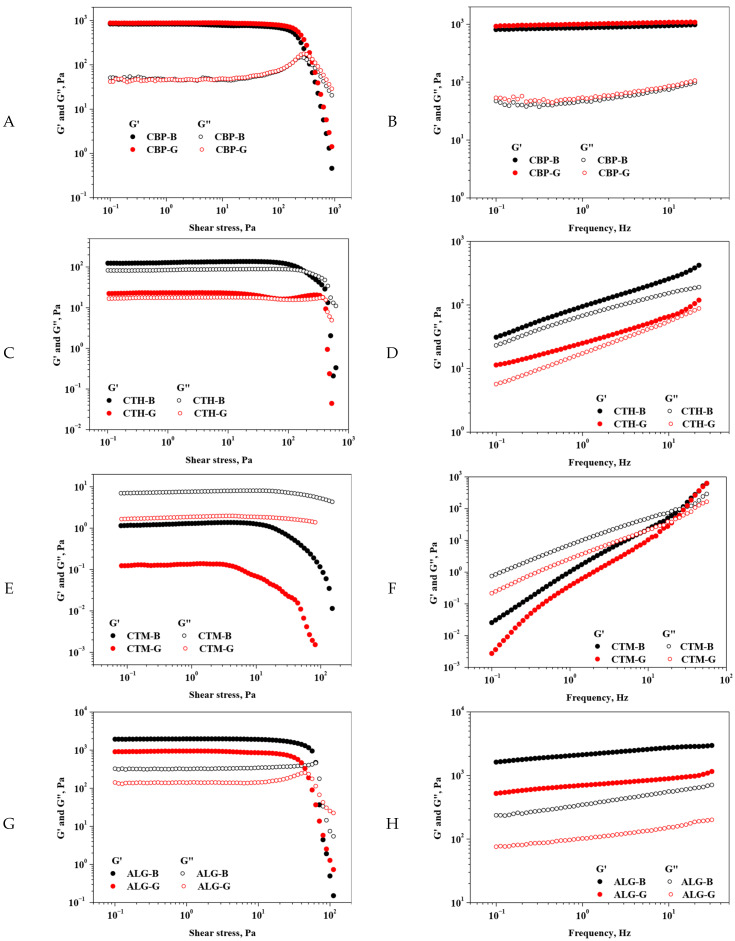
Variation in rheological moduli with shear stress ((**A**). CBP; (**C**). CTH; (**E**). CTM; (**G**). ALG) and frequency ((**B**). CBP; (**D**). CTH; (**F**). CTM; (**H**). ALG). *Legend:* CTH-G—chitosan high-molecular-weight gel; CTM-G—medium-molecular-weight gel; ALG-G—sodium alginate gel; CBP-G—Carbopol 940 gel; CTH-B—chitosan high-molecular-weight gel base; CTM-B—medium-molecular-weight gel base; ALG-B—sodium alginate gel base; CBP-B—Carbopol 940 gel base.

The rheological behaviour of the new gels and their bases was analysed, and the obtained data are depicted in Figure 1.

The CBP-B and CBP-G samples (Figure 1A) show a similar behaviour, where the elastic modulus (G’) and the viscous modulus (G”) have a well-defined viscoelastic region, after which we observe a decrease in the moduli. This decrease represents the irreversible deformation of the CBP-B and CBP-G structures and the appearance of the yield stress point. The determined yield stress points were ~350 Pa for CBP-B and ~400 Pa for CBP-G. Both the critical point and rheological modulus values were slightly improved by the presence of additional ingredients in the CBP-G system formulation. Moreover, the frequency sweep tests (Figure 1B) showed that both the CBP-B and CBP-G samples behave like a gel; G’ is larger than G” with minimal variations in the elastic modulus concerning the applied frequency, which suggests that they have stable, gel-like network structures.

The rheological behaviour of the CBP-B and CBP-G systems aligns with previous findings on Carbopol-based gels. Similar viscoelastic profiles with a G′ > G″ and minor frequency dependency have been reported for Carbopol 940 and 974 systems, indicating strong gel networks with thixotropic recovery and defined structural breakdown at yield stress thresholds [34,35].

The improved moduli and critical point in the CBP-G variant are consistent with observations from Carbopol–polymer composite systems, where additives such as glycerin and hyaluronic acid enhance structural integrity [36,37]. Rheological analysis of CTH-B and CTH-G systems (Figure 1C,D) showed that although G’ is slightly larger than G”, it is still very close in value to G”, indicating that both materials are near a transition between solid and liquid behaviour. The observed trend can be attributed to weak network interactions and structural rearrangements that reduce the difference between the moduli. In addition, the CTH-B sample consistently displays higher G’ and G” values than CTH-G over the entire range of the shear stress and frequencies studied, indicating a stronger structural network.

This marginal dominance of elastic behaviour is indicative of a weak and easily rearrangeable network [38]. Moreover, our observation that CTH-B had a higher G′ and G″ than CTH-G throughout the tests suggests structural reinforcement in the absence of additives, supporting the similar conclusions by Zhao et al. on how dilution or component interference may reduce the rigidity of chitosan networks [39].

A dominant fluid character with detectable LVER but no yield stress points was observed for the CTM-B and CTM-G systems in the sweep amplitude tests (Figure 1E). Moreover, both CTM-based samples showed a predominantly viscous character (G” > G’) at low frequencies and a more elastic behaviour (G’ > G”) at high frequencies. This phenomenon suggests that CTM-B and CTM-G flow like viscoelastic liquids, but when applying high frequencies, they bounce like viscoelastic solids. The crossover frequencies (where G’ equals G”) found for the samples were about 28 Hz for CTM-B and about 22 Hz for CTM-G, which relates to the longest time it takes for the polymer matrix to relax and untangle. CTM-G has a lower crossover frequency point, which means that the polymer matrix relaxes more quickly and flows more easily under stress.

The crossover frequencies (~28 Hz for CTM-B; ~22 Hz for CTM-G) reflect the materials’ transition times between elastic and viscous dominance. These viscoelastic properties are commonly seen in chitosan systems with reduced molecular weight, as lower chain length promotes mobility and flow [40,41,42].

The ALG-B and ALG-G samples (Figure 1H) exhibit a short LVER where G’ is larger than G”, indicating solid-like behaviour. By increasing shear stress, the two rheological moduli tend to decrease, marking the yield stress point. The found yielding points were about 65 Pa for ALG-B and 45 Pa for ALG-G. Therefore, the absence of additional ingredients in the ALG-B formulation resulted in a slight improvement in both the critical point and rheological modulus values. The frequency sweep test (Figure 1H) also indicated that both ALG-B and ALG-G behave like gels. The G’ is larger than G”, and there are only minor changes in the elastic modulus with frequency variation. This result suggests that ALG-based samples have relatively stable and gel-like network topologies. In addition, across the frequency range, ALG-B exhibits consistently greater G’ and loss modulus G” values compared to ALG-G, indicating a stronger gel-like structure with enhanced elasticity and resistance to deformation.

These rheological profiles are consistent with studies highlighting that ionically crosslinked alginate gels tend to exhibit lower yield stress compared to synthetic or covalently crosslinked gels [43]. The stronger performance of ALG-B suggests that the absence of additional additives preserved a more robust gel network, in line with the conclusions drawn by Chan et al., who demonstrated that additive-free alginate systems often retain superior elasticity and consistency due to uninterrupted ionic crosslinking between guluronic acid blocks and calcium ions [43,44,45].

#### 2.2.3. Thermogravimetric Differential Thermal Analysis (TG-DTA)

**Figure 2:**
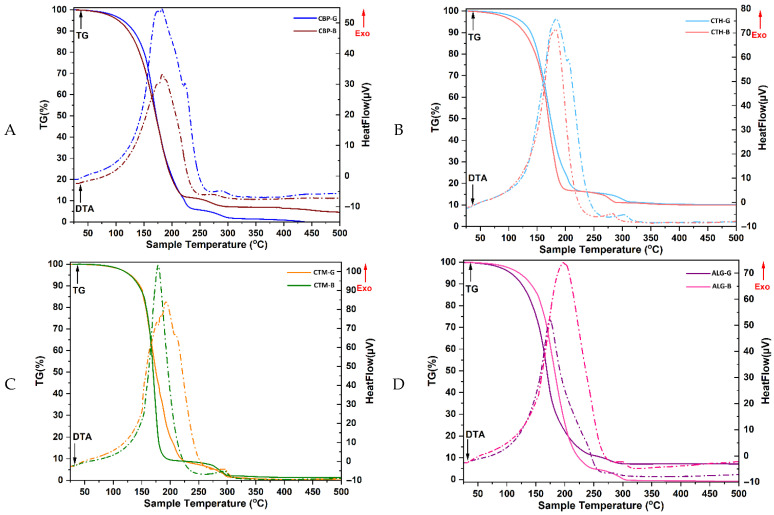
The TG-DTA curves of the gels. (**A**) CBP-G—Carbopol 940 gel and CBP-B—Carbopol 940 gel base; (**B**) CTH-G—chitosan high-molecular-weight gel and CTH-B—chitosan high-molecular-weight gel base; (**C**) CTM-G—medium-molecular-weight gel and CTM-B—chitosan medium-molecular-weight gel base; (**D**) ALG-G—sodium alginate gel and ALG-B—sodium alginate gel base.

The TG-DTA curves obtained for both the hydrogel formulations, and their corresponding bases (Figure 2) showed a consistent two-step thermal degradation pattern, characteristic of multicomponent hydrogel systems. In all cases, the exothermic events observed in the DTA profiles corresponded with the mass loss steps indicated by the TG data.

The first degradation stage, between approximately 100 and 250 °C, is attributed primarily to the evaporation of free and bound water, as well as the evaporation of low-molecular-weight components such as glycerin, ethanol residues from the tinctures, and essential oil constituents. CBP-G exhibited a 94% mass loss up to 230 °C, compared to 90% for CBP-B, indicating a more pronounced release of volatiles in the presence of encapsulated actives. This behaviour is consistent with prior reports, which have associated the first thermal event in Carbopol-based gels with the loss of water and volatile compounds, and the second event with polymer backbone decomposition. The high weight loss observed in the first step suggests that these formulations may retain substantial bound water or volatile actives, especially relevant if essential oils are encapsulated within the matrix [17,46].

In contrast, chitosan-based systems demonstrated increased thermal resistance during this stage. The CTH-G and CTH-B formulations remained thermally stable up to 230 °C and 205 °C, respectively, with initial mass losses of ~10–15%, which reflects the release of water and glycerin. CTM-G and CTM-B samples began degrading slightly around 115 °C and recorded ~10% mass loss up to 230 °C and 200 °C, respectively, consistent with the lower thermal stability of medium-MW chitosan and the influence of actives. The obtained results are also consistent with the thermal behaviour of high-MW chitosan observed in the literature. CTM-G and CTM-B showed a slightly earlier onset of degradation (from ~115 °C), and their overall thermal degradation followed a comparable path [47,48,49].

The ALG-G and ALG-B samples showed rapid degradation starting at 100 °C, with weight losses of ~90% and 95%, respectively, up to 250 °C, due to the highly hydrophilic nature of alginate and the presence of volatile actives. The sodium alginate gels degrade in two significant steps—first corresponding to moisture and low-molecular-weight component loss, and second to polysaccharide decomposition [50,51]. The slightly higher thermal stability of ALG-G (vs. ALG-B) may be associated with differences in crosslinking density or interactions with bioactive encapsulates [52,53,54].

The second degradation stage, extending from ~230 °C to 500 °C, corresponds to the thermal decomposition of the primary polymeric matrix (Carbopol, chitosan, or alginate), as well as the breakdown of *β*-cyclodextrin complexes and thermolabile organic compounds. In this stage, CBP-G decomposed completely by 500 °C, while CBP-B reached a total mass loss of ~95%. CTH-G and CTH-B lost an additional 75–80% of their mass during this interval, resulting in total weight losses of ~90%, reflecting the degradation of the chitosan network. CTM-based samples followed a similar pattern, with near-complete decomposition by 500 °C. ALG-G and ALG-B also underwent complete degradation, reaching total mass losses of ~93% and nearly 100%, respectively.

Overall, these findings confirm that the observed two-step thermal decomposition is governed by both the physical evaporation of volatiles and the chemical degradation of matrix components. Differences in mass loss percentages and onset temperatures between base- and active-loaded formulations highlight the impact of the bioactive ingredients and crosslinking interactions on the thermal behaviour of the gels.

#### 2.2.4. Scanning Electron Microscopy (SEM) Analysis

**Figure 3:**
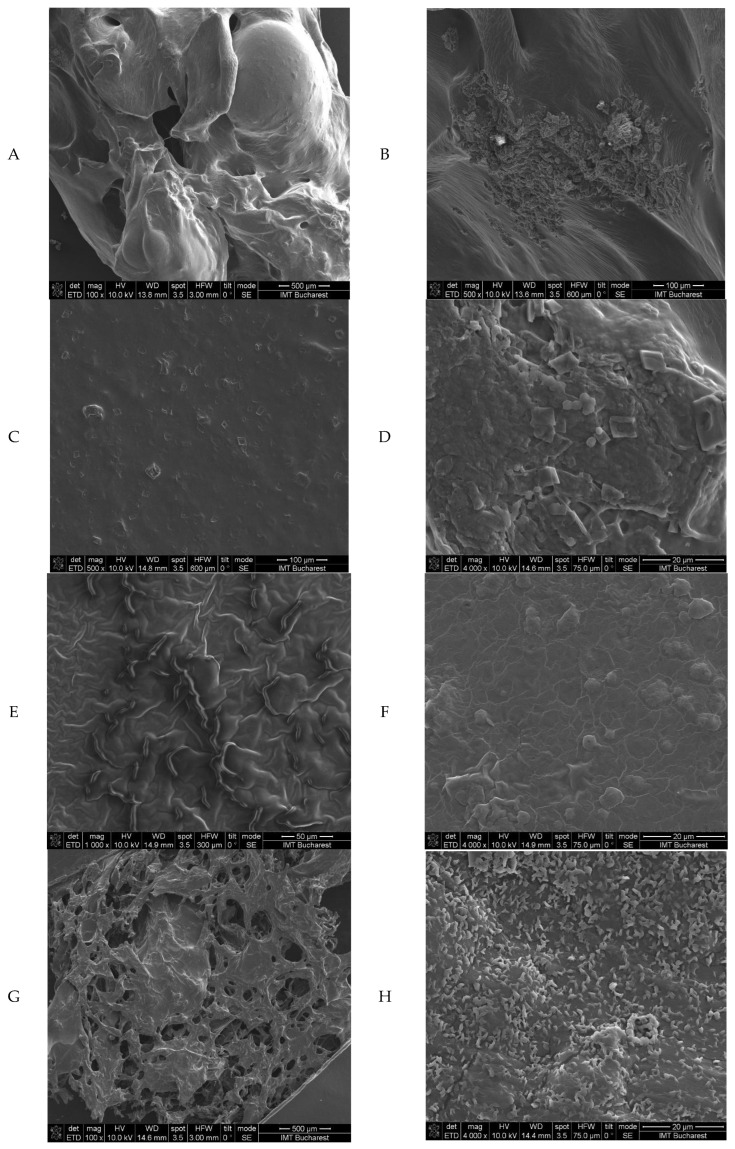
SEM images for the freeze-dried gels: CBP-G at 100× magnification (**A**) and 500× magnification (**B**); CTH-G—chitosan high-molecular-weight gel at 500× magnification (**C**) and 4000× magnification (**D**); CTM-G—chitosan medium-molecular-weight gel at 1000× magnification (**E**) and 4000× magnification (**F**); and ALG-G—sodium alginate gel at 100× magnification (**G**) and 4000× magnification (**H**).

Scanning electron microscopy (SEM) was used to obtain detailed images of the microstructure of the four hydrogel formulations, specifically observing the surface morphology and distribution of active compounds in the hydrogel matrix. Their structural characteristics are important for determining their stability and texture as products intended for topical administration. The SEM images of the gels are presented in Figure 3.

These SEM images (Figure 3) highlight the microarchitecture of the gels, emphasising the uniformity and homogeneity of the gel matrix. The uniform distribution of the active compounds is essential for maintaining the stability of the active ingredients, as it prevents their premature release and degradation, while the hydrogel network contributes to obtaining a texture that allows easy and consistent application on the skin. In addition to these, the gel must keep both its shape and resistance to deformation, key aspects when it comes to maintaining its rheological properties over time and the effective delivery of active ingredients, while the appropriate texture promotes ease of application.

Additionally, the internal organisation of the gels facilitates sustained release of cover compounds (necessary to maintain long-term antimicrobial activity), increasing therapeutic efficacy while decreasing the risk of side effects related to rapid release of active ingredients; hence, the structural properties of the resulting gels (homogeneous distribution, matrix stability, and texture), all favour their use in topical formulations that are more stable, effective for delivery of active compounds, and favourable for therapeutic compliance.

#### 2.2.5. FTIR Analysis Results

**Figure 4:**
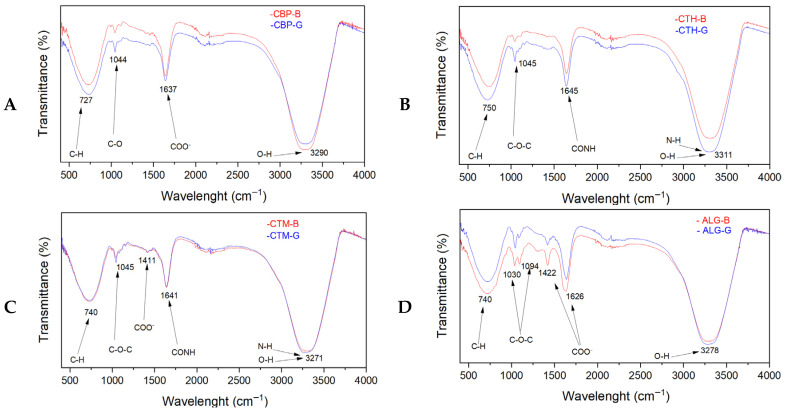
FTIR spectra of CBP-B and CBP-G gel systems (**A**); CTH-B and CTH-G gel systems (**B**); CTM-B and CTM-G (**C**); and ALG-B and ALG-G (**D**).

The FTIR spectra of the analysed samples are presented in Figure 4. The FTIR spectra of the investigated gel formulations provide insight into the interactions between the polymeric matrices (Carbopol; chitosan high molecular weight, CTH; chitosan medium molecular weight, CTM; and alginate) and the incorporated bioactive compounds from plant extracts, essential oils, and the beta-cyclodextrin–clove oil inclusion complex.

In the FTIR spectra of the Carbopol-based gel formulations (CBP-B and CBP-G) (Figure 4A), the following characteristic absorption bands were observed in both gel samples: an absorption band centred at approximately 1637 cm^−1^, attributed in the case of the base formulation, CBP-B, to the asymmetric stretching vibration of carboxylate group (COO^−^) from the Carbopol structure, together with contributions from bound water deformation [17]. In the CBP-G gel formulation, the same band exhibited a slight broadening and intensity increase, suggesting the superposition of additional vibrational modes attributed to the C=C stretching vibrations of aromatic compounds from the plant extracts and eugenol from the *β*-cyclodextrin–clove oil complex, as well as possible hydrogen bonding interactions between the bioactive constituents and the polymer matrix. The absorption band around 3290 cm^−1^ from both the CBP-B and CBP-G gel formulations corresponds to O-H stretching vibrations, indicative of hydrogen bonding present in Carbopol, glycerine, and water compounds. The absorption band observed at 1044 cm^−1^ is assigned to C-O stretching vibrations from hydroxyl groups from glycerine and the polysaccharide backbone of Carbopol. The absorption band near 727 cm^−1^ is attributed to C-H bending vibrations within the polymer structure. Although CBP-G gel formulation contains additional components such as plant extracts, bay leaf oil, and a beta-cyclodextrin–clove oil complex, no significant differences were observed in the IR spectra compared to the CBP-B gel formulation. This aspect is likely due to the relatively low concentrations of these additives, in which case the FTIR spectra show an overlapping of their functional groups with those already present in the Carbopol matrix, and the potential molecular interactions that induce the broadening of the band rather than distinct new peaks. Consequently, the FTIR spectra reflect only the structural features of the Carbopol compound and glycerine in both gel formulations.

The FTIR spectra of the CTH-based gel formulations (CTH-B and CTH-G, Figure 4B) show characteristic bands at ~3311 cm^−1^ (attributed to O-H and N-H stretching), at ~1645 cm^−1^ (attributed to Amide I band and water bending), at ~1045 cm^−1^ (attributed to C-O-C stretching), and at ~750 cm^−1^ (attributed to aromatic C-H bending) [55]. Compared to the CTH-B formulation, the CTH-G gel formulation displayed a slight broadening and intensity increase in these bands, suggesting the formation of additional hydrogen bonding interactions between the chitosan compound and the incorporated plant extracts, essential oils, and *β*-cyclodextrin–clove oil complex, particularly phenolic compounds. The appearance of the band at 750 cm^−1^ also in the CTH-G gel formulation further confirms the successful incorporation of aromatic compounds from essential oils and plant extracts. No significant structural changes were observed in the HMW chitosan polymeric structure upon gel formulation.

The FTIR spectra of the MMW chitosan-based gel formulations (CTM-B and CTM-G, Figure 4C) show characteristic bands at 3271 cm^−1^ (attributed to O-H and N-H stretching), at 1641 cm^−1^ (attributed to Amide I and water bending), at 1411 cm^−1^ (attributed to CH_2_ bending and COO^−^ symmetric stretching), at 1045 cm^−1^ (attributed to C-O-C stretching), and at 740 cm^−1^ (attributed to aromatic C-H bending) [56]. No significant differences in the band position, width, or intensity were observed between the CTM-G and the CTM-B gel formulations, which suggests that weak interactions between the MMW chitosan matrix and the incorporated bioactive compounds are present. Consequently, the MMW chitosan structure remains unaffected by the addition of bioactive components, with no significant spectral modifications. Therefore, the bioactive components (plant extracts, essential oils, and *β*-cyclodextrin–clove oil complexes) are most likely physically embedded within the gel matrix without significant chemical interaction with the polymer structure.

The alginate-based gel formulations (Figure 4D) exhibited characteristic bands at ~3278 cm^−1^ (O-H stretching), ~1626 cm^−1^ (asymmetric COO^−^ stretching), ~1422 cm^−1^ (symmetric COO^−^ stretching), ~1315 cm^−1^ (C-H bending), ~1094 and 1030 cm^−1^ (C-O-C stretching), and ~715 cm^−1^ (aromatic C-H bending). Like the MMW chitosan system, only minor differences in intensity were observed between the base and gel formulations, without any significant shifts or broadening of the absorption bands. These slight intensity variations suggest minimal interactions between the alginate matrix and the bioactive compounds, with the latter predominantly retained via physical entrapment within the hydrogel network, while the polymer backbone remains structurally unaltered.

For alginate-based gel formulations, FTIR spectra exhibit characteristic bands at 1615 cm^−1^ and 1420 cm^−1^, corresponding to asymmetric and symmetric stretching vibrations of carboxylate (COO^−^) groups [57]. The broad absorption band at 3278 cm^−1^ attributed to O-H stretching was more pronounced in the ALG-G gel formulation, showing its higher hydroxyl group content due to the incorporation of the bioactive compounds. The bands at 1315 cm^−1^ are attributed to C-H bending, at 1094 and 1030 cm^−1^ are attributed to C-O-C stretching, and at 715 cm^−1^ are attributed to aromatic C-H bending. Because there were no substantial changes in the FTIR spectra of the ALG-B and ALG-G gel formulations, there are no interactions between the alginate matrix and the incorporated plant extracts, essential oils, and *β*-cyclodextrin–clove oil complex, considering that the bioactive molecules are most likely physically entrapped within the gel network.

Overall, the FTIR analysis indicates the successful incorporation of plant-derived bioactive compounds, essential oils, and the inclusion complex into the gel matrices. Also, the FTIR analysis demonstrates that interactions between the polymer and bioactive compounds are dependent on the nature of the polymeric matrix. The Carbopol and HMW chitosan matrices exhibited more pronounced physical interactions with the incorporated compounds, while the MMW chitosan and alginate matrices demonstrated limited potential for interaction. All around, predominantly in all gel formulations, was the physical encapsulation of the active compounds.

### 2.3. Analysis of Antimicrobial Activity

#### 2.3.1. Qualitative Assessment of the Antimicrobial Activity

Antimicrobial activity was qualitatively evaluated by determining the growth inhibition zone diameters (GIZDs) that appeared around the spot (Table S4).

Qualitative testing of antimicrobial activity revealed a variable effect of all formulations on the tested strains. The chitosan-based gel generated the largest zones of inhibition, an anticipated result due to the intrinsic antimicrobial properties of chitosan [58]. In the case of Gram-negative strains, the Carbopol gel and partly the alginate gel did not show measurable zones of inhibition, which is why additional quantitative tests were performed to evaluate the antimicrobial activity against these strains precisely.

#### 2.3.2. Quantitative Assessment of the Antimicrobial Activity

**Figure 5:**
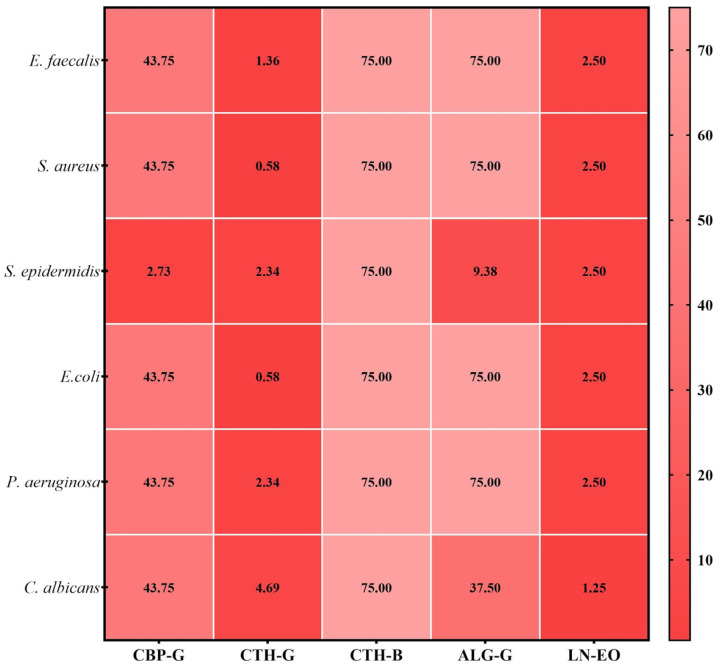
Heatmap of MIC values (mg/mL) for gels. CBP-G–Carbopol 940 gel; CTH-G—chitosan high-molecular-weight gel; ALG-G—sodium alginate gel; CTM-B—chitosan medium-molecular-weight gel base; LN-EO—*Laurus nobilis* essential oil.

The quantitative evaluation of antimicrobial activity was carried out by determining the minimum inhibitory concentration (MIC), defined as the lowest concentration of the antimicrobial agent that inhibits visible microbial growth. The obtained MIC values are illustrated in Figure 5.

The minimum inhibitory concentrations (MICs) determined by quantitative testing ranged from 0.58 to 75 mg/mL. According to the qualitative screening results, the chitosan-based gel showed the most potent antimicrobial activity against all tested strains, especially against *S. aureus* and *E. coli* (MIC = 2.34 mg/mL). For the Carbopol gel, the most effective inhibition was observed against *S. epidermidis* (2.73 mg/mL), like the alginate gel, but at a higher concentration (9.38 mg/mL). *Laurus nobilis* essential oil showed notable antimicrobial activity on *C. albicans* (1.25 mg/mL), with slightly weaker effects on the other strains (2.50 mg/mL). No antimicrobial activity was recorded for the Carbopol and alginate bases.

#### 2.3.3. Semiquantitative Assessment of the Microbial Adherence to the Inert Substratum

**Figure 6:**
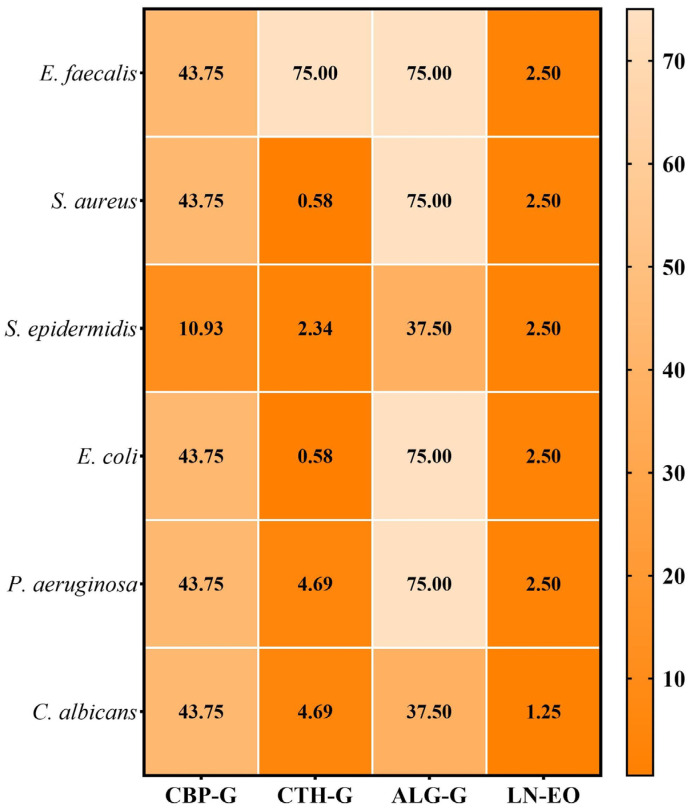
Heatmap of MBEC values (mg/mL) for gels. CBP-G—Carbopol 940 gel; CTH-G—chitosan high-molecular-weight gel; ALG-G—sodium alginate gel; LN-EO—*Laurus nobilis* essential oil.

The MBEC results are presented in Figure 6.

The minimum biofilm eradication concentration (MBEC) is an essential parameter in assessing the efficacy of antimicrobials, especially in the context of biofilm-associated infections, which are notoriously difficult to treat. Biofilms give microorganisms additional protection, increasing their resistance compared to planktonic forms. By evaluating the MBEC, this study provides relevant data on the effectiveness of these formulations under clinical-like conditions, demonstrating their ability to act on both free cells and biofilm-organised forms.

Following the assessment of microbial adhesion on an inert substrate, MBEC values ranged from 0.58 to 75.0 mg/mL. As expected, the best results were obtained for the chitosan-based gel, with the lowest MBEC values recorded for *S. aureus* and *E. coli* strains, consistent with previous MIC data. The alginate gel showed the lowest antibiofilm efficacy, with MBEC values ranging from 37.5 to 75.0 mg/mL.

The demonstrated antimicrobial activity of the chitosan-based gel is due not only to the active components in its composition, but also to the presence of chitosan itself. Chitosan adds significant intrinsic antimicrobial activity to the gel due to its cationic properties. Protonation of amine groups in an acidic environment confers its polycationic character, facilitating electrostatic interaction with the cell wall, resulting in disruption of integrity and bacterial lysis. This effect is not exclusively dependent on the concentration of other components but is directly embedded in the mechanism of action of chitosan [59,60].

Literature data show that chitosan-based gels exhibit significantly better MICs/MBCs; for example, standard chitosan gels generated larger diameter zones of growth inhibition for *S. aureus*, with a reported MIC value of 16 µg/mL [59].

In the study by Goy et al., (2015), a concentration of 1 g/L chitosan resulted in a 96% reduction in the growth of *S. aureus* after 12 h compared to the control. At the same time, chitosan solutions at 0.5–2 g/L also clearly reduced the proliferation of *E. coli*, although to a slightly lesser extent than Gram-positives. This difference in efficiency suggests that chitosan, in its polycationic form and with an optimal molecular weight, is more effective against Gram-positive bacteria, likely due to the thicker and more negatively charged peptidoglycan layer of their cell walls [60].

Chitosan not only inhibits growth but also affects biofilm integrity, a significant barrier to the treatment of persistent infections. Its cationic structure enables the elimination of biofilms by inhibiting the adhesion and penetration of proteins that stabilise the microbial communities. Thus, chitosan-based gels can guarantee not only the release of active compounds but also an independent curative antimicrobial effect, enhancing the final results [61].

In the case of the VAT and ATT, antimicrobial activity was demonstrated against standard strains of *S. aureus*, *B. subtilis*, *P. aeruginosa*, *E. coli*, and *C. albicans*, as can be found in a previously published study. The minimum inhibitory concentration (MIC) values varied between 15.63 and 500 µg extract/mL, the lowest values recorded for the tinctures, especially, on a significant number of strains. The results obtained can be attributed to the higher content of phenolic acids and flavonoids in these extracts, compared to the other extracts. Regarding their antibiofilm activity, the best results were obtained for the VAT, with the lowest MBEC values recorded for both tinctures studied [32]

Other literature data show that the methanolic extract (and essential oil) of *A. triphylla* exhibits a potent antimicrobial activity against *E. coli* and *B. subtilis*, as well as a relevant antifungal effect on several strains of *Candida*, including *C. albicans* ATCC 2091, *C. glabrata* ATCC, and *C. parapsilosis* [62]. Also, the antifungal activity was confirmed against some strains isolated from clinical samples, such as *C. albicans*, *C. dubliniensis*, *C. glabrata*, *C. krusei*, *C. guillermondii*, *C. parapsilosis*, and *C. tropicalis*. In the case of *V. officinalis*, most research indicates a significant inhibitory effect on *S. aureus*, *P. aeruginosa*, *E. coli*, *S. typhi*, *C. freundii*, and *B. subtilis* [63,64,65].

*Laurus nobilis* essential oil also exhibits significant antimicrobial activity due to its composition, rich in 1,8-cineole and α-terpinyl acetate. It has been shown to be effective against the Gram-negative bacteria *E. coli*, *P. aeruginosa, S. enterica*, and *K. pneumoniae*, as well as against the Gram-positive *B. subtilis*, *S. aureus, E. faecalis*, and *L. monocytogenes* [66,67,68]. Antifungal activity against *Aspergillus* species (*A. clavatus*, *A. niger*) and *Candida* has also been reported according to other studies [69].

The antimicrobial activity of the *β*-cyclodextrin–clove oil complex was evaluated against several bacterial strains, including *Enterococcus faecalis*, *Staphylococcus aureus*, *Bacillus subtilis*, *Escherichia coli*, *Enterobacter cloacae*, and *Pseudomonas aeruginosa*. The lowest MIC value was recorded for *Escherichia coli*, at 0.039 mg/mL, indicating superior antimicrobial efficiency against this strain. In addition, the complex proved effective in inhibiting the formation of bacterial biofilms at a concentration of 0.156 mg/mL, as observed in *Pseudomonas aeruginosa* and *Bacillus subtilis* strains [31].

### 2.4. Cell Viability Assessment

**Figure 7:**
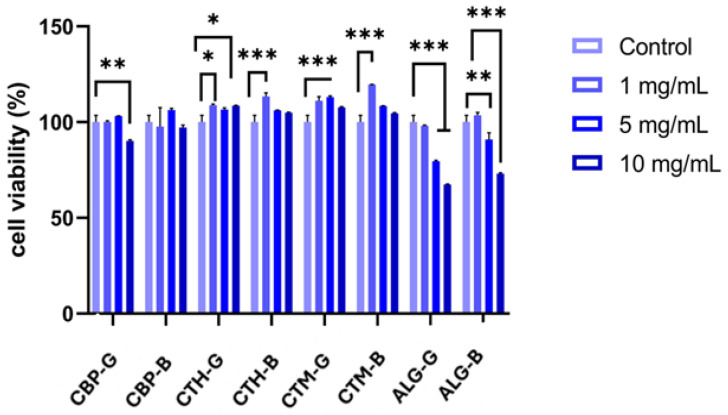
Effect of the gel formulations on the cell viability of HaCaT human keratinocytes after 24 h. Data are expressed as mean ± SD, *n* = 3, and the differences were evaluated using ANOVA. Compared with the control (untreated cells), * *p* < 0.01, ** *p* < 0.01, and *** *p* < 0.001. CBP-G—Carbopol 940 gel; CTH-G—chitosan high-molecular-weight gel; CTM-G—medium-molecular-weight gel; ALG-G—sodium alginate gel; CBP-B—Carbopol 940 gel base; CTH-B—chitosan high-molecular-weight gel base; CTM-B—medium-molecular-weight gel base; ALG-B—sodium alginate gel base.

The MTT assay revealed differential effects of the pharmaceutical formulations on HaCaT cell viability after 24 h of treatment, as shown in Figure 7. Formulations with high-molecular-weight chitosan (CTH-G and CTH-B) and medium-molecular-weight chitosan gels (CTM-G and CTM-B) induced a significant increase in cell viability compared to the control, suggesting a potential proliferative effect, which may be attributed to the presence of chitosan. Conversely, formulations containing alginate exhibited a dose-dependent reduction in viability, with a statistically significant decrease (30% compared to untreated cells) observed at the 5 mg/mL and 10 mg/mL concentrations (Figure 7). These results indicate that higher concentrations of alginate-based gels may exert cytotoxic effects on keratinocytes.

**Figure 8:**
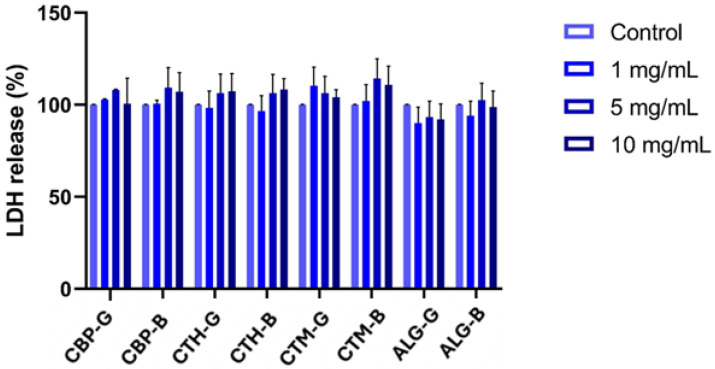
Evaluation of cytotoxicity by LDH assay following exposure of HaCaT human keratinocytes to various concentrations of gels after 24 h. Results are expressed as percentages relative to untreated controls (set at 100%) and are presented as mean ± SD (*n* = 3). CBP-G—Carbopol 940 gel; CTH-G—chitosan high-molecular-weight gel; CTM-G—medium-molecular-weight gel; ALG-G—sodium alginate gel; CBP-B—Carbopol 940 gel base; CTH-B—chitosan high-molecular-weight gel base; CTM-B—medium-molecular-weight gel base; ALG-B—sodium alginate gel base.

Assessment of LDH release showed no statistically significant changes across the tested formulations and concentrations. The levels of extracellular LDH remained comparable to those of the control group, indicating that none of the treatments caused substantial membrane damage or overt cytotoxicity under the conditions tested (Figure 8).

**Figure 9:**
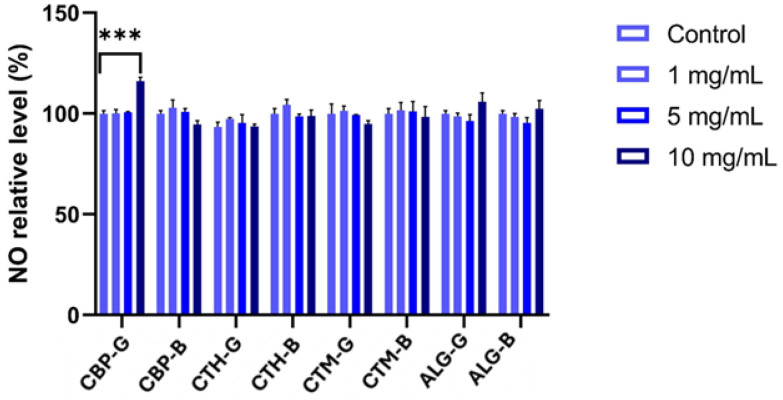
Nitric oxide (NO) production in HaCaT cell line after 24 h exposure to 1, 5, and 10 mg/mL of gels. Results are expressed as percentages relative to untreated controls (set at 100%) and are presented as mean ± SD (*n* = 3). Statistical significance was determined by two-way ANOVA followed by Tukey’s multiple comparisons test: *** *p* < 0.0001. CBP-G—Carbopol 940 gel; CTH-G—chitosan high-molecular-weight gel; CTM-G—medium-molecular-weight gel; ALG-G—sodium alginate gel; CBP-B—Carbopol 940 gel base; CTH-B—chitosan high-molecular-weight gel base; CTM-B—medium-molecular-weight gel base; ALG-B—sodium alginate gel base.

NO production, measured as an indicator of inflammatory response, remained unchanged for many formulations and concentrations. However, a significant increase in nitric oxide levels was detected only at the highest concentration (10 mg/mL) of formulation CG (Figure 9). This may suggest a mild pro-inflammatory effect associated with this specific condition.

The present study evaluated the effects of various pharmaceutical gel formulations on the viability, cytotoxicity, and inflammatory response of HaCaT keratinocytes. The data suggest that the nature of the polymer matrix plays a critical role in modulating cellular behaviour. Formulations that contained chitosan were associated with a significant increase in cell viability (Figure 7). This observation is consistent with previous reports highlighting the biocompatibility and bioactive properties of chitosan, including its ability to promote wound healing and stimulate cell proliferation [70].

Chitosan’s known interaction with cell surface receptors and its role in enhancing growth factor stability may explain the proliferative response observed in HaCaT cells [71].

Formulations CG and CB, which included Carbopol as the gelling agent, did not exhibit a strong proliferative or cytotoxic effect. This suggests that Carbopol-based matrices are mainly inert under the tested conditions, maintaining cell viability comparable to the control. These findings support the use of Carbopol as a neutral vehicle for topical formulations where minimal biological interaction is desired [72,73].

On the other hand, the alginate gel showed a dose-dependent reduction in cell viability, particularly at higher concentrations (5 and 10 mg/mL). Alginate is often considered biocompatible, yet in vitro data suggest that high concentrations or ionic interactions in the matrix can impair cell adhesion and proliferation, particularly in monolayer cultures such as HaCaT cells [74,75].

The observed cytotoxicity may also be related to residual crosslinking agents or osmotic effects.

LDH assay results confirmed the absence of significant membrane damage across all formulations and concentrations, reinforcing the interpretation that most observed changes in viability are likely due to metabolic or proliferation-related effects rather than overt cytotoxicity. Interestingly, nitric oxide (NO) levels remained stable across most treatments, except for the alginate gel at 10 mg/mL, which induced a modest but significant increase in NO production. This effect may reflect a localised inflammatory or stress response, although the magnitude was limited and did not correlate with decreased viability or increased LDH release.

Overall, the data suggest that chitosan-based formulations promote keratinocyte viability, while alginate gels may reduce cell proliferation at higher concentrations. These findings can inform the design of topical formulations intended for wound healing or dermatological applications.

## 3. Conclusions

This study presents a comparative evaluation of topical antimicrobial gels incorporating plant-derived bioactive products—*Laurus nobilis* essential oil, tinctures of *Verbena officinalis* and *Aloysia triphylla*, and a *β*-cyclodextrin–clove essential oil inclusion complex—formulated within four distinct polymer matrices: Carbopol 940, sodium alginate, and chitosan of high and medium molecular weight.

This comparative approach enabled systematic insight into how polymer architecture influences rheology behaviour, antimicrobial efficacy, and cytocompatibility. Among them, HMW chitosan-based gels showed the most promising profile, combining potent antimicrobial activity (low MIC and MBEC values against *S. aureus* and *E. coli*) with enhanced HaCaT cell viability and viscoelastic properties suitable for topical application. CBP-based gels exhibited excellent structural integrity, with high yield points (~400 Pa), thermal stability, and minimal cytotoxicity, making them suitable carriers for sensitive bioactive compounds when low cellular interaction is desired. In contrast, alginate-based gels displayed moderate mechanical stability and dose-dependent cytotoxicity at higher concentrations, suggesting some limitations for direct skin contact in undiluted forms.

The incorporation of plant extracts, essential oils, and *β*-cyclodextrin–clove essential oil complexes in gel bases was successfully achieved, as confirmed by FTIR and SEM analysis. The structural analysis supported the sustained release potential and uniform distribution of the compounds within the gel matrices. Thermogravimetric evaluation confirmed the thermal robustness of all systems.

Overall, the findings support the suitability of chitosan- and Carbopol-based gels for formulating stable, biocompatible, and effective topical delivery systems for natural antimicrobials. These systems offer a promising foundation for further development of dermatological formulations aimed at managing cutaneous infections and promoting wound healing.

This study’s strength lies in demonstrating the possibility of preparing preservative-free pharmaceutical formulations for reduced additive exposure and improved tolerability. The findings align with recent review results, notably a 2025 paper [76], which highlights how novel delivery systems can reduce the volatility and irritation of plant essential oils while simultaneously enhancing their antimicrobial and pharmacological efficacy in topical skincare applications. Future efforts should focus on identifying innovative advanced packaging and novel intrinsic antimicrobial strategies to overcome stability challenges and broaden the applicability of preservative-free drugs across various pharmaceutical forms.

## 4. Materials and Methods

### 4.1. Reagents

All reagents used in the experiments were of analytical purity, purchased from the company Sigma-Aldrich (Merck KGaA; Darmstadt, Germany): chitosan HMW ((C_6_H_11_O_4_N)n; CAS Number: 9012-76-4; molecular weight: >300 kDa), chitosan MMW ((C_6_H_11_O_4_N)n; CAS: Number 9012-76-4; molecular weight: >300 kDa), Sodium alginate ((C_6_H_7_NaO_6_)n; CAS Number: 9005-38-3; molecular weight: 200 kDa), Carbopol 940 ((C_3_H_4_O_2_)_n_; CAS Number: 9003-01-4; molecular weight: 450 kDa), *β*-cyclodextrin (C_42_H_70_O_35_; CAS Number: 7585-39-9; molecular weight: 1134.98), ethanol (C_2_H_6_O; CAS Number: 64-17-5; molecular weight: 46), methanol (CH_4_O; CAS Number: 67-56-1; molecular weight: 32), acetic acid (C_2_H_4_O_2_; CAS Number: 64-19-7; molecular weight: 60.05), hexane (C_6_H_12_; CAS Number: 110-54-3; molecular weight: 84), and calcium chloride (CaCl_2_; CAS Number: 10043-52-4; molecular weight: 110.98 g/mol, mol). *Laurus nobilis* essential oil was purchased from the company Elemental S.R.L.

### 4.2. Plant-Derived Ingredients

#### 4.2.1. The Inclusion Complex’s Preparation and Characterisation

The inclusion complex between *Eugenia caryophyllata* essential oil and *β*-cyclodextrin was prepared as previously reported (kneading method) [31,73]. The thermal stability and encapsulation efficiency of the *β*-cyclodextrin complex were consistent with previously published data [31]. Therefore, the current investigation focused on integrating the complex and extracts into the gel matrices and evaluating their physicochemical and biological performance.

#### 4.2.2. Tincture Preparation and Characterisation

The VAT and ATT were obtained according to previously described protocols [32].

#### 4.2.3. GC-MS Analysis of *Laurus nobilis* Essential Oil

The essential oil of *Laurus nobilis* (sweet bay) was subjected to gas chromatography for analysis. The system under consideration was a Focus gas chromatograph linked to a Polaris Q ion trap mass spectrometer (Thermo Fisher Scientific, Waltham, MA, USA). For the purpose of analysis, the essential oil samples were prepared in hexane at a volume ratio of 1:10. The separation of the mixtures was carried out on a DB-5MS capillary column (25 m × 0.25 mm × 0.25 μm) by means of a Triplus autosampler. Helium was utilised as the carrier gas at a flow rate of 1 mL/min. The initial oven temperature programme was set at 60 °C for a duration of three minutes. After that, the oven temperature was increased at a rate of 10 °C per minute until it reached a maximum of 200 °C. The temperature was further maintained at this level for a period of two minutes. Subsequently, the oven temperature was increased at a rate of 12 °C per minute until it reached a final temperature of 240 °C. The ion source and interface temperatures were 200 °C and 250 °C, respectively. For the mass spectrometry analysis, the instrument was operated in electron impact mode at 70 electronvolts (eV), acquiring data in full-scan mode across an m/z range of 35 to 300. Following this, the chromatograms were analysed, and the compounds were identified using Xcalibur software (version 4.1) and the NIST 11 database. The Kovats indices (KIs) were established by employing an alkane standard solution (C_8_–C_20_ in hexane; Sigma-Aldrich, St. Louis, MO, USA). The composition of the essential oil was expressed as a percentage of each compound, calculated based on their respective GC peak areas within the total chromatogram.

### 4.3. Formulation Design

The objective of this study was to develop and pharmaco-technically characterise, especially in terms of rheology, volatile oil-based hydrogels. For the formulation of gels with antimicrobial action, the variation in the polymer was considered to select the optimal gel formulation for topical use.

The gels’ compositions are presented in Table 1. The gel bases are considered as the formulation without bioactive compounds (VAT, ATT, *Laurus nobilis* essential oil, and *β*-CD-ECEO complex).

The overall experimental workflow is shown in Supplementary Figure S1 (Workflow scheme: from formulation to biological evaluation).

### 4.4. Physical Characterisation Methods

To bring a comprehensive understanding of the physical properties of the hydrogel systems, a set of four techniques was employed. Rheological analysis was used to evaluate the viscoelastic behaviour and mechanical stability, while thermal analysis (TG-DTA) provided insights into their thermal stability and decomposition profile. Scanning electron microscopy (SEM) was employed to examine the microstructure and surface morphology. At the same time, FTIR spectroscopy facilitated the identification of functional groups and confirmed the chemical interactions within the polymeric matrix.

#### 4.4.1. Rheological Analysis

The rheological behaviour of the systems was investigated using the Kinexus Pro rheometer (Worcestershire, UK) connected to a Julabo CF41 cryo-compact circulator (Seelbach, Germany). Geometries with a roughened surface were used, with a lower plate diameter of 50 mm and an upper plate of 40 mm, and the gap was set at 0.6 mm. The linear viscoelastic region (LVER) was determined by applying amplitude sweep stress tests where the applied frequency was 1 Hz. Subsequently, the viscoelastic behaviour was investigated using the frequency sweep test, where the frequency was varied from 0.1 to 20 Hz, and the shear stress was kept constant (from the LVER). All measurements were recorded at 25 °C.

#### 4.4.2. Thermal Analysis Procedure (TG-DTA)

The thermogravimetric differential thermal analysis (TG-DTA) of the hydrogels was carried out with a Themys One 1150 TGA (New Castle, DE, USA) in a nitrogen atmosphere. This study was carried out in Pt crucibles and heated from 30 °C up to 500 °C at a heating rate of 20 °C/min.

#### 4.4.3. Scanning Electron Microscopy Procedure

Surface morphology of the samples was obtained using an FEI Nova NanoSEM 630 scanning electron microscope with a Through-Lens-Detector (Nanotech, Tokyo, Japan) at an acceleration voltage of 5 kV and a working distance of 4.7 mm.

#### 4.4.4. FTIR Characterisation

Fourier-transform infrared (FTIR) spectra were acquired using a JASCO FT/IR 4700 spectrophotometer (Tokyo, Japan) equipped with a monolithic diamond attenuated total reflectance (ATR) accessory (Tokyo, Japan). Measurements were performed over a wavenumber range of 400–4000 cm^−1^ at an incident angle of 45°, with 64 scans averaged at a resolution of 4 cm^−1^.

### 4.5. Antimicrobial Activity

Antimicrobial evaluations were conducted using the following standard reference strains: *Enterococcus faecalis* ATCC 29212, *Staphylococcus aureus* ATCC 25923, *Staphylococcus epidermidis* ATCC (strain number incomplete), *Escherichia coli* ATCC 25922, *Pseudomonas aeruginosa* ATCC 27853, and *Candida albicans* ATCC 10231. All microbial strains were obtained from the Microorganism Collection of the Department of Microbiology, Faculty of Biology, and the Research Institute of the University of Bucharest.

*Laurus nobilis* essential oil was diluted in DMSO at 20 mg/mL for testing. The DMSO was also evaluated to confirm that it did not exhibit antimicrobial activity against the tested bacterial strains.

#### 4.5.1. Qualitative Assay of the Antimicrobial Activity

The antimicrobial activity of the gels was evaluated using the spot diffusion method, following the guidelines established by the Clinical Laboratory Standards Institute [73,77,78]. Bacterial and yeast suspensions, adjusted to 1.5 × 10^8^ CFU/mL and 3 × 10^8^ CFU/mL respectively, were prepared from 24-h cultures grown on Nutrient Broth No. 2 (NB) and Sabouraud Glucose Agar supplemented with chloramphenicol (Sab). The prepared suspensions were used to inoculate Petri dishes containing the corresponding culture media, after which 20 µL of each gel sample was applied by spotting onto the surface. Sterile medium served as the negative control, while the positive control consisted of NB or Sab medium inoculated with the respective microbial suspensions. Following application, the plates were incubated at 37 °C for 24 h for bacterial strains and 48 h for yeast strains. 

#### 4.5.2. Quantitative Assay of the Antimicrobial Activity

The minimum inhibitory concentration (MIC) assessment was performed using an adapted binary serial microdilution standard assay in liquid media utilising 96-well microtiter plates. From each sample, serial two-fold microdilutions were realised in 150 µL of the corresponding broth medium seeded with the standard inoculum. The microtiter plates were incubated at 37 °C for 24 h. Visual and spectrophotometric analyses determined the MIC values by measuring the absorbance at 620 nm via the BIOTEK SYNERGY-HTX ELISA multi-mode reader (Winooski, VT, USA) [73,77].

#### 4.5.3. Semiquantitative Assay of the Microbial Adherence to the Inert Substratum

Biofilm formation on the inert substrate was evaluated using the same serial two-fold microdilution method. After 24 h of incubation, the media from the microtiter plates (containing the binary sample dilutions) were carefully removed, and the wells were washed three times with sterile phosphate-buffered saline to eliminate non-adherent cells. The biofilm-adherent bacteria were then fixed with methanol for 5 min and stained with 1% crystal violet for 15 min. Subsequently, the stained biofilm was solubilised using 33% acetic acid, and the absorbance was measured at 490 nm using a spectrophotometric plate reader [73,77].

### 4.6. Experimental Protocol for Viability Assay

HaCaT human keratinocyte cells were cultured in high-glucose Dulbecco’s Modified Eagle Medium (DMEM), supplemented with 2 mM L-glutamine and 10% foetal bovine serum (FBS). Cells were maintained in a humidified incubator at 37 °C with 5% CO_2_. For experiments, cells were seeded in 24-well plates at a density of 1 × 10^5^ cells/mL. After cell adhesion, they were treated with pharmaceutical formulations at concentrations of 1 mg/mL, 5 mg/mL, and 10 mg/mL for 24 h. Untreated cells served as negative control. Following treatment, assays were performed to assess cell viability, cytotoxicity, and inflammatory response indicators.

Cell viability was assessed using a colorimetric MTT assay (3-(4, 5-dimethylthiazol-2-yl)-2, 5-diphenyltetrazolium bromide), as described by Mosmann (1983) [79].

After treatment, the culture medium was removed, and the cells were gently washed with phosphate-buffered saline (PBS). Subsequently, 500 µL of MTT solution (1 mg/mL) was added to each well and incubated for 2 h at 37 °C. The MTT solution was then discarded, and the resulting formazan crystals were dissolved in 100% isopropanol. Absorbance was measured at 570 nm using a FlexStation 3 Multi-Mode Microplate Reader (Molecular Devices LLC, San Jose, CA, USA).

Cytotoxicity was quantified by measuring the release of lactate dehydrogenase (LDH) into the culture medium, an indicator of membrane integrity. The assay was performed according to the manufacturer’s protocol [Cytotoxicity Detection Kit (LDH) (Roche, Basel, Switzerland)]. After 24 h of treatment, a volume of 50 μL of culture medium from treated and untreated cells was mixed with 50 μL of reaction mixture (catalyst-to-dying solution ratio of 1:45). Then, the cells were incubated in the dark at room temperature for 15 min and the absorbance was subsequently measured at 490 nm using a FlexStation 3 Multi-Mode Microplate reader.

The production of nitric oxide (NO) was evaluated in the culture supernatant 24 h after treatment. A volume of 80 µL from each sample was mixed in a 1:1 ratio with the Griess reagent (a mixture of sulfanilamide and N-(1-naphthyl) ethylenediamine dihydrochloride). After incubation at room temperature, absorbance was measured at 540 nm using the FlexStation 3 Multi-Mode Microplate Reader and analysed with SoftMax Pro software (version 7.1.2.1) (Molecular Devices LLC, San Jose, CA, USA).

### 4.7. Statistical Analysis

The experiments were performed in triplicate. Statistical analyses were conducted using GraphPad Prism (version 8; GraphPad Software, La Jolla, CA, USA). Data are expressed as mean ± SD, *n* = 3, and the differences were evaluated using two-way ANOVA, followed by Tukey’s multiple comparisons test to assess the significance between the treated samples and controls. Statistical significance was considered at *p*  <  0.05 (*), *p*  <  0.01 (**), and *p* <  0.001 (***).

## Figures and Tables

**Table 1:** Gel compositions.

Formulation	Polymer	Active Ingredients			Plasticizer	Gelling Agent	Solvent
		Tinctures	Essential Oil	Other			
	2%	2%	1%	0.50%	5%	1%	Until 100%
**CBP-G**	Carbopol 940	*Verbena officinalis* (VAT) *Aloysia triphylla* (ATT)	*Laurus nobilis*	*β*-CD-ECEO complex	Glycerin	NA	Purified water
**CTH-G**	Chitosan HMW						
**CTM-G**	Chitosan MMW						
**ALG-G**	Sodium alginate					CaCl_2_	

*Legend:* Chitosan HMW—chitosan high molecular weight; Chitosan MMW—chitosan medium molecular weight; *β*-CD-ECEO complex—*β* cyclodextrin–*Eugenia caryophyllata* essential oil complex; NA—not applicable.

## Data Availability

The original contributions presented in this study are included in the article; further inquiries can be directed to the corresponding author.

## Supplementary Materials

The following supporting information can be downloaded at https://www.mdpi.com/article/10.3390/gels11080653/s1, Table S1: The chemical composition of *Laurus nobilis* essential oil; Table S2: New gel characteristics; Table S3: Gel base characteristics; Table S4: The influence of developed pharmaceutical formulations on selected pathogenic strains; Figure S1: Workflow scheme: from formulation to biological evaluation.
