# Supplementary material for: New Antimicrobial Gels Based on Clove Essential Oil–Cyclodextrin Complex and Plant Extracts for Topical Use
Source: Gels. 2025 Aug 18;11(8):653. doi: 10.3390/gels11080653 (PMC12386016; doi:10.3390/gels11080653)
Supplement: Supplementary file 1 [file gels-11-00653-s001.zip › gels-3781003-supplementary.pdf]

gels

Supplementary Information

New Antimicrobial Gels Based on Clove Essential Oil–Cyclodextrin Complex and Plant Extracts for Topical Use

Table S1. The chemical composition of *Laurus nobilis* essential oil.

Compounds	KI*	Relative area (%)
α-Pinene	923	4.23±0.27
Camphene	937	0.29±0.01
Sabinene	964	8.65±0.49
β-Pinene	980	0.48±0.04
2,3-Dehydro-1,8-cineole	996	0.25±0.00
α-Phellandrene	1003	0.04±0.00
α-Terpinene	1010	0.01±0.00
Eucalyptol	1028	60.65±1.09
trans-β-Ocimene ((E)-β-Ocimene)	1036	0.22±0.01
α-Terpinene	1049	0.89±0.04
cis-Sabinene hydrate	1060	0.10±0.00
α-Terpinolene	1078	0.29±0.00
Linalool	1087	0.70±0.03
cis-*p*-Menth-2-en-1-ol	1112	0.13±0.00
trans-Pinocarveol	1132	0.28±0.00
δ-Terpineol	1159	0.42±0.01
Terpinen-4-ol	1170	2.90±0.14
α-Terpineol	1182	1.81±0.09
Myrtenol	1189	0.21±0.01
4-thujen-2-yl acetate	1260	0.10±0.00
Bornyl acetate	1275	0.29±0.01
p-Cymen-7-ol	1287	0.07±0.00
Terpinen-4-ol-acetate	1303	0.54±0.03
α-Terpinyl acetate	1337	11.17±0.75
Eugenol	1344	0.63±0.03
α-Ylangene	1360	0.22±0.01
α-Copaene	1377	0.40±0.02
Methyleugenol	1385	1.15±0.06
β-Caryophyllene	1409	0.47±0.02
α-Guaiene	1430	0.06±0.00
γ-Muurolene	1480	0.24±0.00
Germacrene D	1484	0.19±0.00
α-Muurolene	1504	0.15±0.01
γ-Cadinene	1512	0.19±0.01
δ-Cadinene	1525	0.15±0.01

Spathulenol	1575	0.15±0.01
Caryophyllene oxide	1582	0.19±0.01
γ-Eudesmol	1630	0.23±0.01
Total		99.15

(*KI-Kovats Index)

Table S2. New Gels characteristics.

Characteristic	CBP-G	CTH-G	CTM-G	ALG-G
**Initial macroscopic characteristics**	appearance: homogenous; color: green-brown; smell: specific	appearance: homogenous; color: green-brown; smell: specific	appearance: homogenous; color: green-brown; smell: specific	appearance: homogenous; color: green-brown; smell: specific
**Macroscopic characteristics after 90 days**	appearance: homogenous; color: green-brown smell: specific	appearance: homogenous; color: green-brown; smell: specific	appearance: homogenous; color: green-brown; smell: specific	appearance: homogenous; color: green-brown; smell: specific

Legend: CTH-G- Chitosan high molecular weight gel, CTM-G- medium molecular weight gel, ALG-G- sodium alginate gel, CBP-G- Carbopol 940 gel

Table S3. Gels bases characteristics.

Characteristic	CBP-B	CTH-B	CTM-B	ALG-B
**Initial macroscopic characteristics**	appearance: homogenous; color: clear to white; smell: without smell	appearance: homogenous; color: yellowish; smell: without smell	appearance: homogenous; color: yellowish; smell: without smell	appearance: homogenous; color: clear to white; smell: without smell
**Macroscopic characteristics after 90 days**	appearance: homogenous; color: clear to white smell: without smell	appearance: homogenous; color: yellowish; smell: without smell	appearance: homogenous; color: yellowish; smell: without smell	appearance: homogenous; color: clear to white; smell: without smell

Legend: CTH-B- Chitosan high molecular weight gel base, CTM-B- medium molecular weight gel base, ALG-B- sodium alginate gel base, CBP-B- Carbopol 940 gel base.

Table S4. The influence of developed pharmaceutical formulations on selected pathogenic strains

Strains	Samples							
	CBP-G	CTH-G	ALG-G	CBP-B	CTH-B	ALG-B	LN-EO	DMSO
**Gram-positive bacteria**								
E. faecalis ATCC 29212	+	+++	+	-	++	-	+	-
S. aureus ATCC 25923	+-	+++	+-	+-	++	+-	++	-
S. epidemidis ATCC	+-	+++	+++	+-	++	+-	++	-
**Gram-negative bacteria**								
E. coli ATCC 25922	++	+++	++	-	++	+-	++	-
P. aeruginosa ATCC 27853	++	+++	++	+	++	+	++	-
**Yeasts**								
C. albicans ATCC 10231	+	++	+-	-	+	+-	++	-

Legend: CBP-G- Carbopol 940 gel, CTH-G- Chitosan high molecular weight gel, ALG-G- sodium alginate gel, CBP-B- Carbopol 940 gel base, CTH-B- Chitosan high molecular weight gel base, CTM-B- medium molecular weight gel base, ALG-B- sodium alginate gel base, LN-EO- *Laurus nobilis* essential oil, DMSO- dimethyl sulfoxide. (-)/ pale yellow is represented by the absence of

the inhibition; (+-)/ peach is represented by growth inhibition for which the diameter of the inhibition zone couldn't be measured (+)/ light orange = GIZD ≤ 5 mm; (++)/ orange = GIZD between 5 and 10 mm; (+++)/ red = GIZD between 10 and 18 mm.

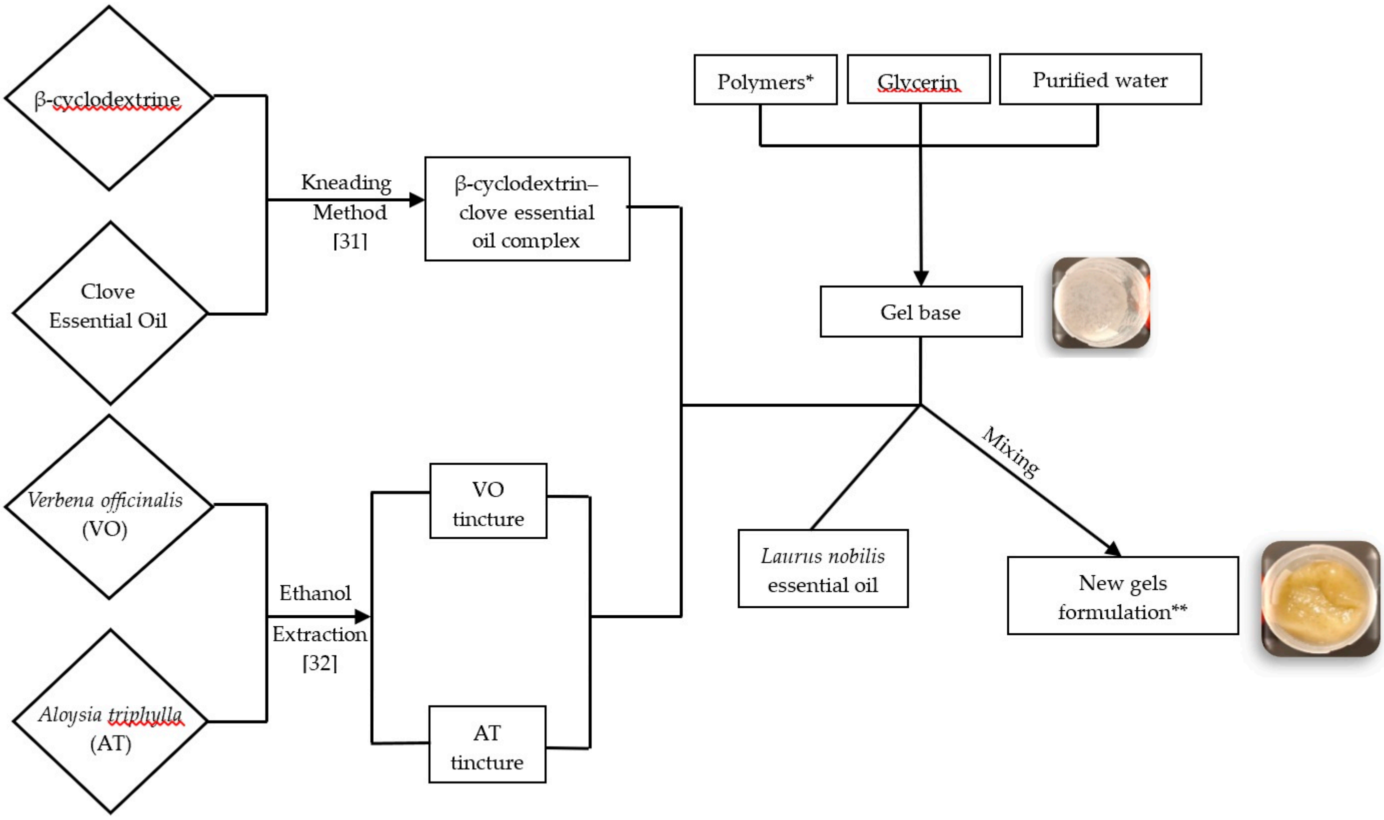

Figure S1. Workflow Scheme: From Formulation to Biological Evaluation
*Polymers: Carbopol 940, Sodium Alginate, Chitosane HMW, Chitosane MMW
**New gels formulation: CBP-G, ALG-G, CTH-G, CTM-G

References

[31]: Stancu, A.I.; Mititelu, M.; Ficai, A.; Ditu, L.-M.; Buleandră, M.; Badea, I.A.; Pincu, E.; Stoian, M.C.; Brîncoveanu, O.; Boldeiu, A.; et al. Comparative Evaluation of β-Cyclodextrin Inclusion Complexes with Eugenol, Eucalyptol, and Clove Essential Oil: Characterisation and Antimicrobial Activity Assessment for Pharmaceutical Applications. Pharmaceutics 2025, 17, 852, doi:10.3390/pharmaceutics17070852.

[32]: Stancu, A.-I.; Geană, E.I.; Ditu, L.-M.; Ficai, A.; Nagoda, E.; Oprea, E. Chemical Composition and Antimicrobial Activity of Verbena Officinalis and Aloysia Citrodora Extracts Obtained by Traditional and Laboratory Methods. U.P.B. Sci. Bull., Series B 2024, 86, 111–122.
